# Functional Role of Long-Chain Acyl-CoA Synthetases in Plant Development and Stress Responses

**DOI:** 10.3389/fpls.2021.640996

**Published:** 2021-03-22

**Authors:** Huayan Zhao, Dylan K. Kosma, Shiyou Lü

**Affiliations:** ^1^State Key Laboratory of Biocatalysis and Enzyme Engineering, School of Life Sciences, Hubei University, Wuhan, China; ^2^Department of Biochemistry and Molecular Biology, University of Nevada, Reno, Reno, NV, United States

**Keywords:** long-chain acyl-CoA synthetases, lipid, biosynthesis, regulation, stress, metabolism

## Abstract

Fatty acids (FAs) play vital roles in plants as components of lipid membranes that demarcate cells and organelles, as sources of stored energy in the form of neutral lipids, and as signaling molecules that elicit plant responses to adverse conditions. The activation of FAs through the formation of acyl-CoA intermediates by acyl-CoA synthetase (ACS) family enzymes is required for their synthesis and degradation. Long-chain ACSs (LACSs) represent a small subgroup of ACS enzymes that specifically convert long-chain or very-long-chain FAs into corresponding thioesters for multiple lipid-associated processes. Alteration of LACS activity often results in pleiotropic phenotypes such as male sterility, organ fusion, aberrant cuticular structure, delayed seed germination, altered seed oil content, and plant capacity to respond to various environmental stresses. This review provides a comprehensive analysis of LACS family enzymes including substrate specificity, tissue-specific expression patterns, and distinct subcellular localization highlighting their specific roles in lipid synthesis and degradation, the effects of altered LACS activity on plant development, the relationship between LACS activity and stress resistance, and the regulation of LACS activity. Finally, we pose several major questions to be addressed, which would advance our current understanding of LACS function in plants.

## Introduction

Fatty acids (FAs) are nearly ubiquitous in plant cells where they are incorporated into various types of lipids including phospholipids and other membrane lipids, triacylglycerols (TAGs), as well as epidermal cuticular lipids and suberin, and thus are important for maintaining membrane integrity, providing energy for various metabolic processes, and forming surface barriers against abiotic and biotic stresses (Fulda et al., 2004; Schnurr et al., 2004; Li-Beisson et al., 2013; Grevengoed et al., 2014; Jessen et al., 2015; Fich et al., 2016; Ingram and Nawrath, 2017). The shunting of FAs into different metabolic pathways initially requires activation to high-energy CoA thioesters by the activity of acyl-CoA synthetases (ACSs), also known as FA:CoA ligases. The formation of acyl-CoAs mediated by ACS is an ATP-dependent process that proceeds as a two-step reaction. In the first step, the α-phosphorous of ATP is attacked by the partially negatively charged oxygen on the carbonyl on an FA forming an acyl-AMP intermediate and releasing pyrophosphate. In the second step, the thiol group of CoA conducts a nucleophilic attack on the carbonyl of the acyl-AMP, forming a fatty acyl-CoA and releasing AMP (Grevengoed et al., 2014).

Long-chain ACSs (LACSs) as a subgroup of ACS family preferentially activate long-chain (LCFAs; C16–C18) or very-long-chain FAs (VLCFAs; ≥C20) (Shockey et al., 2002; Lü et al., 2009; Shockey and Browse, 2011). Here, LACS enzymes differ in terms of substrate specificity, tissue-specific expression pattern, and subcellular distribution, confining each member to specific metabolic pathways. Furthermore, since LACS activity is closely related with production traits such as oil quality and quantity, and stress resistance, in recent years, research on LACS has also been carried out in economically relevant crop species. Herein, we present a concise review of progress in understanding LACS function in plants and further pose major unanswered questions about LACS enzymes that need to be addressed in order to deepen our understanding of these critical enzymes.

## Substrate Preference

Despite being a small subgroup of ACS enzymes in *Arabidopsis*, AtLACSs show a surprising diversity in substrate specificity albeit restricted to LCFAs or VLCFAs (Shockey et al., 2002). AtLACS isoform divergence in substrate specificity is easily related to their diversity in function and differential subcellular localizations (Table 1). AtLACS1, a major isoform involved in the synthesis of wax, activates VLCFAs (20:0–30:0) with the highest specificity for triacontanoic acid (30:0) (Lü et al., 2009) (Table 1). AtLACS1 shows the next highest activity with palmitic acid (16:0). In agreement with this, mutants with loss of function of *AtLACS1* have substantial reductions in 16:0-derived cutin monomers (Lü et al., 2009). AtLACS2 is mainly responsible for cutin synthesis, preferring hydroxylated over unsubstituted FAs, with enhanced activity toward hydroxylated 16:0 (Schnurr et al., 2004) (Table 1). AtLACS4, AtLACS8, and AtLACS9 appear to play redundant roles in wax and cutin synthesis, showing catalytic activities with LCFAs similar to those of AtLACS1 and AtLACS2 but displayed lower activities with hydroxylated versus unsubstituted FAs (Shockey et al., 2002; Fulda et al., 2004; Zhao et al., 2019). Additionally, simultaneous mutation of AtLACS4 and AtLACS9 led to a significant reduction of linoleic acid (18:2) at the *sn2* position of MGDG, indicating that 18:2 might also be a preferred substrate of AtLACS4 or AtLACS9 (Jessen et al., 2015). Peroxisome-localized AtLACS6 and AtLACS7 mediate TAG degradation during seed germination, activating FAs stored in seed TAGs with enhanced activity for eicosenoic acid (20:1), which is predominantly found in *Arabidopsis* seed lipids (Table 1) (Fulda et al., 2002, 2004; Shockey et al., 2002).

**TABLE 1:** Substrate specificity, subcellular localization, and function of LACSs.

Species	Protein	Substrate specificity	Subcellular localization	Function	References
*Arabidopsis thaliana*	AtLACS1	16:0, 16:1, 18:1,18:2, 20:0 (Shockey et al., 2002); 16:0, 18:0, 20:0, 22:0, 24:0, 26:0, 28:0, 30:0↑ (Lü et al., 2009)	ER	Wax, cutin, TAG, and tryphine	Lü et al., 2009; Zhao et al., 2010, 2019; Yang et al., 2017
	AtLACS2	16:0, 16:1, 18:1,18:2 (Shockey et al., 2002); 16:0, 16-OH16:0↑ (Schnurr et al., 2004); 16:0, 18:0, 20:0, 22:0, 24:0, 26:0, 28:0, 30:0 (Lü et al., 2009)	ER	Cutin, Wax, TAG, related with hypoxia response	Schnurr et al., 2004; Suh et al., 2005; Lü et al., 2009; Yang et al., 2017; Zhao et al., 2019
	AtLACS3	16:0, 16:1↑, 18:1,18:2	Unidentified	Unidentified	Shockey et al., 2002
	AtLACS4	16:0, 16:1, 18:1,18:2↑ (Shockey et al., 2002)	ER	Wax, TAG, tryphine, and glycerolipid	Zhao et al., 2010, 2019; Jessen et al., 2011
	AtLACS5	16:0, 16:1↑, 18:1, 18:2 (Shockey et al., 2002)	Unidentified	Unidentified	Shockey et al., 2002
	AtLACS6	16:0, 16:1, 18:1, 18:2, 20:1↑ (Shockey et al., 2002); 14:0, 16:0, 18:1,18:2, 18:3	Peroxisome	β-oxidation	Fulda et al., 2002
	AtLACS7	16:0, 16:1, 18:1, 18:2, 20:1↑ (Shockey et al., 2002); 14:0, 16:0, 18:1↑, 18:2, 18:3, 20:1 (Fulda et al., 2002)	Peroxisome	β-oxidation	Fulda et al., 2002
	AtLACS8	16:0, 16:1, 18:1,18:2	ER	Wax and TAG	Shockey et al., 2002; Zhao et al., 2010, 2019
	AtLACS9	16:0, 16:1, 18:1,18:2	Plastid envelop	Wax, TAG, and glycerolipid	Shockey et al., 2002; Zhao et al., 2010, 2019
*Brassica napus*	BnLACS2	14:0, 16:0, 18:0, 18:1, 22:1	ER	TAG	Ding et al., 2020
*Glycine max*	GmACSL2	14:0, 16:0, 18:0, 18:1, 22:1	Peroxisome	β-oxidation	Yu et al., 2014
*Gossypium hirsutum*	GhACS1	14:0, 16:0, 18:0, 18:1, 20:0	ER, PM	Microsporogenesis	Wang and Li, 2009
*Helianthus annuus*	HaLACS1	16:1, 18:1, 18:2, 18:3	Plastid envelop	Unidentified	Aznar-Moreno et al., 2014
	HaLACS2	16:0, 16:1, 18:1,18:2	ER	Unidentified	Aznar-Moreno et al., 2014
*Malus domestica*	MdLACS2	Unidentified	Unidentified	Wax	Zhang et al., 2020a
	MdLACS4	Unidentified	Unidentified	Wax	Zhang et al., 2020b
*Oryza sativa*	OsLACS9	Unidentified	Plastid envelope	Starch degradation	Kitajima-Koga et al., 2020
*Thalassiosira pseudonana*	TpLACSA	16:0, 18:3, 18:4, 20:4↑, 20:5↑, 22:6↑	Unidentified	TAG	Tonon et al., 2005
*Zea mays*	ZmCER8	Unidentified	Unidentified	Wax	Zheng et al., 2019
*Linum usitatissimum*	LuLACS8A	18:1, 18:2, 18:3↑	Unidentified	TAG	Xu et al., 2018
*Ricinus communis*	RcACS2	12:0, 16:0, 18:1, 18:2, 18:3, 12-OH18:1↑, 11-OH12:0, 12-OH12:0	Unidentified	TAG	He et al., 2007
	RcACS4		Unidentified	Unidentified	

*Arrows indicate the enhanced activity. LACSs, long-chain acyl-CoA synthetases; ER, endoplasmic reticulum; TAG, triacylglycerol.*

The substrate specificity of the LACS enzymes has been extensively studied in plant species besides *Arabidopsis*. They also preferentially utilize LCFAs or VLCFAs. For example, GhACS1 from cotton (*Gossypium hirsutum*), sharing high amino acid sequence identity with AtLACS4 and AtLACS5, is required for normal microsporogenesis (Wang and Li, 2009). *In vitro* enzyme activity analysis revealed that GhACS1 prefers LCFAs with the highest activity toward oleic acid (18:1) (Wang and Li, 2009). MdLACS2 from apple (*Malus domestica*), an ortholog of AtLACS2, was shown to catalyze the formation of 16:0 CoA (Zhang et al., 2020a). BnLACS2 from rapeseed (*Brassica napus*), involved in seed oil production, exhibited a substrate preference for 14:0, 16:0, 18:0, 18:1, and 22:1 (Ding et al., 2020). These studies demonstrate that LACS enzymes play conserved roles in higher plants. However, given that lipid metabolic processes vary among different plant species, LACS proteins will exhibit species-dependent substrate specificities. For example, α-linolenic acid (18:3) was enriched in flax (*Linum usitatissimum*) seed oil; recently, LuLACS8A from flax was identified to show high activity toward 18:3 (Xu et al., 2018). RcACS2 from *Ricinus communis* is found to preferentially activate ricinoleic acid (12-OH 18:1), which accumulates to very high levels in castor oil (He et al., 2007). TpLACSA in *Thalassiosira pseudonana* preferentially catalyzes the formation of several molecular species of polyunsaturated VLCFA CoAs (Tonon et al., 2005) (Table 1).

## Expression Pattern

**FIGURE 1:**
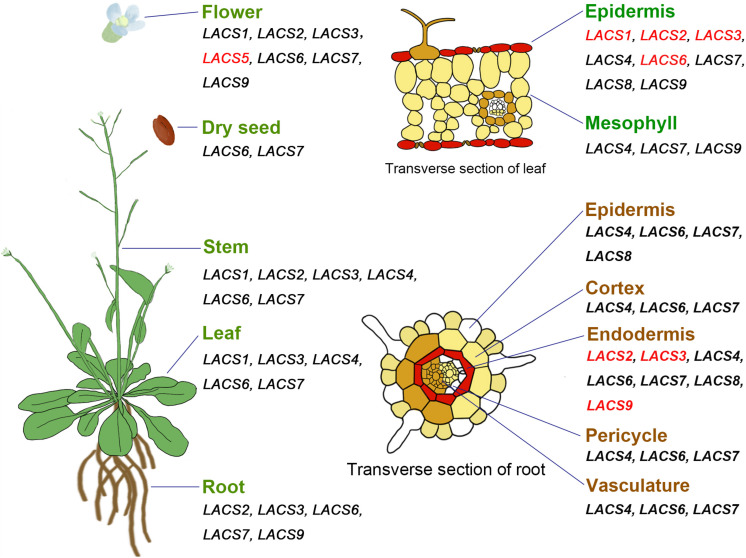
Expression pattern of *AtLACS* genes in different organs or tissues. All genes are from *Arabidopsis thaliana*. Genes specifically expressed in certain organs or tissues are marked with red color.

In *Arabidopsis*, most *AtLACS* genes show broad expression patterns (Figure 1); i.e., they are highly expressed in multiple organs including root, stem, leaf, flower, and germinating seedlings (Shockey et al., 2002; Zhao et al., 2010). Different from these *AtLACS* genes, *AtLACS3* is only expressed in root, stem, leaf, and flower, whereas *AtLACS5* is exclusively detected in flower (Shockey et al., 2002). Moreover, analysis of *Arabidopsis* eFP Browser data^1^ reveals that some *AtLACS* genes exhibit tissue-specific expression patterns. For example, *AtLACS1*, *AtLACS2*, *AtLACS3*, and *AtLACS6* are specifically expressed in leaf epidermal cells where cuticular lipids are produced; and *AtLACS2*, *AtLACS3*, and *AtLACS9* are highly expressed in root endodermal cells where suberin is synthesized (Figure 1), suggestive of their possible roles in apoplastic lipid synthesis in these tissues.

The expression pattern of *LACS* genes has also been extensively investigated in other plant species such as *B. napus* and *M. domestica*, *Helianthus annuus*, and *Glycine max* (Table 2). A total of 34 *BnLACSs* genes are found in the *B. napus* genome, 18 of which are expressed in developing seeds (Xiao et al., 2019), implicating major roles for these genes in lipid metabolism. These *BnLACS* genes show diversified expression patterns. Several genes display broad expression patterns including two *BnLACS4s*, two *BnLACS8s*, and one *BnLACS9*, quite similar to their closest *Arabidopsis* orthologs (Xiao et al., 2019), whereas some genes show tissue-specific expression patterns. For example, several *BnLACS5* genes are specifically expressed in buds, anthers, and stamens (Xiao et al., 2019), resembling *AtLACS5*. One cotton ortholog, *GhACS1*, also displays an expression pattern *similar to AtLACS5*, indicating the conserved function of these *LACS5s* in flower development (Wang and Li, 2009). Another research group also comprehensively checked the expression of a number of *MdLACS* genes in apple and found that all tested genes are highly expressed in pericarp tissues where wax and cutin are actively produced (Zhang et al., 2018), suggesting their possible roles in cuticle synthesis.

**TABLE 2:** Expression pattern of *LACS* genes from different plants.

Species	Gene name	Gene ID	Expression pattern	References
*Brassica napus*	*BnLACS1-2*	BnaAnng39590D	Highly expressed in leaves and buds	Xiao et al., 2019
	*BnLACS1-3*	BnaC04g51420D	Expressed in all organs except for roots, highly expressed in leaves and buds, siliques, and pericarps	
	*BnLACS1-9*	BnaCnng60230D	Highly expressed in leaves, moderately expressed in flowers and buds	
	*BnLACS1-10*	BnaA05g00640D	Highly expressed in buds, moderately expressed in flowers	
	*BnLACS2-1/BnLACS2*	BnaA05g16170D	Highly expressed in buds and developing seeds, moderately expressed in leaves and flowers, lowly expressed in roots and stems	Xiao et al., 2019; Ding et al., 2020
	*BnLACS2-2*	BnaC05g51350D	Highly expressed in buds	Xiao et al., 2019
	*BnLACS4-1*	BnaA01g13470D	Expressed in all organs, highly expressed in leaves and flowers	
	*BnLACS4-2*	BnaC01g15670D	Expressed in all organs, highly expressed in leaves and flowers	
	*BnLACS5-1*	BnaC09g26090D	Highly expressed in buds, anthers, and stamens	
	*BnLACS5-2*	BnaA09g22150D	Highly expressed in buds, anthers, and stamens	
	*BnLACS5-3*	BnaC02g28920D	Highly expressed in buds, anthers, and stamens	
	*BnLACS5-4*	BnaA02g21860D	Highly expressed in buds, anthers, and stamens	
	*BnLACS6-1*	BnaC03g34500D	Highly expressed in leaves and flowers	
	*BnLACS6-2*	BnaA03g29320D	Expressed in all organs, highly expressed in leaves and flowers	
	*BnLACS8-1*	BnaC03g44430D	Expressed in all organs, highly expressed in flowers	
	*BnLACS8-2*	BnaA03g57930D	Expressed in all organs, highly expressed in leaves and flowers	
	*BnLACS9-3*	BnaC06g20910D	Highly expressed in buds, moderately expressed in flowers	
	*BnLACS9-4*	BnaA07g20920D	Highly expressed in buds	
*Malus domestica*	*MdLACS1*	MD09G1286100	Highly expressed in pericarps, moderately expressed in stems	Zhang et al., 2018
	*MdLACS2.1*	MD05G1070800	Highly expressed in pericarps	
	*MdLACS2.2/MdLACS*	MD10G1085400	Expressed in all organs, highly expressed in pericarps and young leaves, moderately expressed in roots, stems, flowers, fruits, seeds, sarcocarps, and climax leaves	Zhang et al., 2018, 2020a
	*MdLACS4.1*	MD06G1106100	Highly expressed in pericarps	Zhang et al., 2018
	*MdLACS4.2/MdLACS4*	MD14G1128200	Expressed in all tissues, highly expressed in pericarps, lowly expressed in roots, stems, young leaves, mature leaves, flowers, fruits, seeds, and sarcocarps	Zhang et al., 2018, 2020b
	*MdLACS6.1*	MD13G1188800	Expressed in all tissues, highly expressed in pericarps, climax leaves, and stamens	Zhang et al., 2018
	*MdLACS6.2*	MD16G1189300	Expressed in all tissues, highly expressed in pericarps	
	*MdLACS8.1*	MD09G1129700	Highly expressed in pericarps, climax leaves, and stamens	
	*MdLACS8.2*	MD17G1118500	Highly expressed in pericarps, moderately expressed in stems, seeds, sarcocarps and climax leaves	
	*MdLACS9.1*	MD08G1163100	Highly expressed in pericarps and climax leaves	
	*MdLACS9.2*	MD15G1349800	Expressed in all tissues, highly expressed in pericarps	
*Gossypium hirsutum*	*GhACS1*	DQ174259	Highly expressed in anther	Wang and Li, 2009
	*GhACS2*	DQ174260		
*Glycine max*	*GmACSL2*	Glyma_05G151200	Highly expressed in germinating seedlings and young leaves, moderately expressed in developing seeds and flowers, lowly expressed in roots, stems, and senescent leaves	Yu et al., 2014
*Oryza sativa*	*OsLACS9*	Os12g0102350	Highly expressed in shoots, moderately expressed in leaves	Ding et al., 2020
*Helianthus annuus*	*HaLACS1*	HM490305	Highly expressed in seeds	Aznar-Moreno et al., 2014
	*HaLACS2*	HM490306	Highly expressed in cotyledons, stems, and leaves	
*Ricinus communis*	*RcACS2*	DQ300358	Highly expressed in germinating seeds	He et al., 2007
	*RcACS4*	DQ300359	Ubiquitously expressed in all organs	

## Subcellular Localization

**FIGURE 2:**
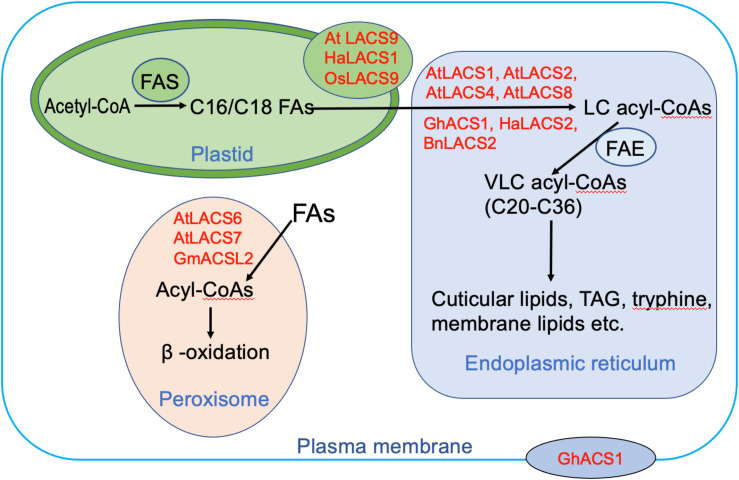
The possible roles of long-chain acyl-CoA synthetases (LACSs) residing in different subcellular compartments. The proteins shown here are from *Arabidopsis thaliana* (*At*), *Brassica napus* (*Bn*), *Gossypium hirsutum* (*Gh*), *Helianthus annuus* (*Ha*), and *Oryza sativa* (Os). Abbreviations: FAS, fatty acid synthesis complex; FAE, fatty acid elongation complex; TAG, triacylglycerol; LCFA, long-chain fatty acid; VLCFA, very-long-chain fatty acid.

To minimize futile cycles, LACS orthologs in mammals are distributed across many different cellular compartments such as plasma membrane (PM), lipid droplet, mitochondria, peroxisome, and endoplasmic reticulum (ER) (Soupene and Kuypers, 2008). In plants, the LACS proteins are confined to fewer subcellular compartments including ER, plastid, peroxisome, and PM (Figure 2). Plastids are the places where *de novo* synthesis of FAs occurs. Several LACS orthologs are known to localize to the plastid envelope (Figure 2), including AtLACS9, HaLACS1 from sunflower (*H. annuus*), and OsLACS9 from rice (*Oryza sativa*) (Schnurr et al., 2002; Aznar-Moreno et al., 2014; Jessen et al., 2015; Kitajima-Koga et al., 2020). They are implicated to function in the activation of *de novo* synthesized LCFAs in plastids. Moreover, AtLACS9 is also identified to be involved in lipid trafficking between plastid and ER (Jessen et al., 2015). ER is the compartment where membrane lipids, cuticular lipids, and TAG are produced. Most identified AtLACSs localize to this organelle including AtLACS1, AtLACS2, AtLACS4, and AtLACS8 (Figure 2). Similarly, one AtLACS2 ortholog from *B. napus* known for its roles in rapeseed oil synthesis, BnLACS2, is also found in the ER (Ding et al., 2020). Surprisingly, GhACS1 from cotton (*G. hirsutum*), despite sharing high amino acid sequence identity with AtLACS4 and AtLACS5, displays different localization patterns being found not only in ER but also in PM, implicating dual roles in two compartments (Wang and Li, 2009). Distinct from the LACS proteins involved in lipid synthesis, LACS proteins mediating FA degradation usually localize to peroxisomes. To date, three LACS proteins are found to reside in peroxisome, including AtLACS6, AtLACS7, and one soybean protein GmACSL2 (Fulda et al., 2002, 2004; Yu et al., 2014). Their localization often requires peroxisomal targeting signal (PTS) sequences. For instance, AtLACS6 localization is targeted by a type 2 PTS (PTS2), whereas AtLACS7 localization is driven by both PTS1 and PTS2 (Fulda et al., 2002). Moreover, AtLACS6, AtLACS7, and GmACSL2 have been identified to be involved in lipid degradation, providing energy for seedling development (Fulda et al., 2004; Yu et al., 2014). Taken together, it can be surmised that LACSs residing in different subcellular compartments play distinct roles in lipid synthesis and degradation. We found it intriguing that despite sharing high sequence identity with each other, LACS proteins sometimes display different subcellular expression patterns. For example, the dual localization of GhACS1 to ER and PM is different from that of its *Arabidopsis* ortholog AtLACS4, suggesting overlapping but also distinct functions for these LACS orthologs in different species. Further study of LACS in diverse plant species is required to comprehensively clarify the function of *LACS* genes during lipid metabolism.

## Long-Chain Acs Associated With Cuticular Lipid Biosynthesis

Cuticle mainly consists of cutin and wax. Cutin is a polyester mainly composed of glycerol and C16/C18 FA derivatives (Fich et al., 2016). Wax is composed of VLCFAs and corresponding derivatives in *Arabidopsis* (Lee and Suh, 2013). The synthesis of cutin and wax requires LACS activity. Cutin biosynthesis starts in plastid where *de novo* FA synthesis occurs, and then the generated LCFAs are transported from plastid to ER for final modification. During this process, LACSs facilitate the formation of LCFA-CoAs prior to the further modification of LCFA for integration into the cutin polymer (Fich et al., 2016). *AtLACS2* is identified to be essential for this process. Its mutation resulted in drastic reduction of dicarboxylate cutin monomers, predominant components of *Arabidopsis* cutin (Bessire et al., 2007), and a significant reduction of total stem cutin loads, suggesting a predominant role for *AtLACS2* in cutin synthesis under normal growth conditions. In addition, a recent study reported that the *AtLACS2* activity also affects plant sensitivity to submergence by changing cuticle permeability (Xie et al., 2020). AtLACS1, sharing high amino acid sequence similarity with AtLACS2, is also involved in cutin synthesis, but its role is subordinate (Lü et al., 2009; Weng et al., 2010). Simultaneous suppression of AtLACS1 and AtLACS2 activity resulted in more severe cuticle defects than observed in either parental mutant, signifying their overlapping roles for the generation of LCFA-CoA esters (Lü et al., 2009; Weng et al., 2010). Interestingly, our recent study has shown that AtLACS1, rather than AtLACS2, specifically coordinates with CER17/ADS4 for the synthesis of cutin in upper, younger portions of inflorescence stems, demonstrating an organ and developmental-specific function for AtLACS1 in cutin biosynthesis (Yang et al., 2017). Apart from AtLACS1 and AtLACS2, AtLACS4 is also involved in cutin biosynthesis; the total cutin amount of *Arabidopsis lacs1 lacs2 lacs4* triple mutant is much lower than that of either single or double mutants (Zhao et al., 2019), indicative of functional redundancy among these isoforms.

Just like cutin, wax synthesis also proceeds through two processes occurring in two different compartments, i.e., the synthesis of LCFAs in plastid, and the elongation and modification of VLCFAs in ER. LACS catalyzes the binding of CoA with LCFA or VLCFA prior to the modification of VLCFA. Several AtLACSs are identified to play roles in this process, including AtLACS1, AtLACS2, AtLACS4, AtLACS8, and AtLACS9 (Lü et al., 2009; Weng et al., 2010; Zhao et al., 2019), which function redundantly in wax synthesis given that higher-order mutants often possess severer defects in wax accumulation than their parental lines (Lü et al., 2009; Weng et al., 2010; Zhao et al., 2019). Among these proteins, AtLACS1 (also known as CER8) is the main isoform responsible for wax biosynthesis, since decreasing AtLACS1 activity alone leads to a drastic reduction of most wax components including alkanes, aldehydes, 2-alcohols, and ketones, finally resulting in the dramatic reduction of total wax amounts (Lü et al., 2009; Weng et al., 2010).

## Long-Chain Acss Related With Tag Synthesis

Triacylglycerol accumulates to very high levels in developing seeds serving as an energy reserve for seed germination and seedling growth. TAG biosynthesis starts with *de novo* FA synthesis in plastids. Plastid-derived LCFAs are transported into ER and esterified into a glycerol backbone. The activation of LCFAs to CoA thioesters by LACSs provides acyl-CoAs for TAG synthesis. Several AtLACSs are known for their involvement in TAG synthesis. *AtLACS1*, *AtLACS2*, *AtLACS4*, *AtLACS8*, and *AtLACS9* are highly expressed in developing seeds, suggesting their potential role in seed oil production (Zhao et al., 2010). *AtLACS1* and *AtLACS9* were the first two genes identified to be involved in TAG synthesis (Zhao et al., 2010), although AtLACS1 and AtLACS9 are localized in two different compartments (Zhao et al., 2010). Among nine *Arabidopsis* LACS proteins, AtLACS9 is the only isoform residing in the outer membrane of plastids. Its loss of function alone has little effect on TAG synthesis, whereas the introduction of *AtLACS1* defects in an *atlacs9* background causes a moderate reduction of FA content, indicative of overlapping roles in TAG synthesis (Zhao et al., 2010). Besides *AtLACS1* and *AtLACS9*, other *AtLACS* genes highly expressed in seeds include *AtLACS2*, *AtLACS4*, and *AtLACS8*, which are also reported to be involved in TAG synthesis. Decreased oil content was detected in seeds of double mutants including *atlacs1 atlacs2*, *atlacs1 atlacs4*, *atlacs4 atlacs8*, and *atlacs4 atlacs9* (Zhao et al., 2010, 2019). Higher-order mutants show a greater reduction in oil; the TAG content of *atlacs1atlacs2atlacs4* triple mutant seeds is far lower than that of either parental double mutant (Zhao et al., 2019).

Long-chain ACS involvement TAG synthesis has been characterized not only in *Arabidopsis* but also in economically relevant plant species such as *B. napus*. These genes play conserved roles across plant species (Xiao et al., 2019; Ding et al., 2020). Several *LACS* orthologs from *B. napus* are found to be highly expressed in high oil content cultivars (Xiao et al., 2019). Furthermore, overexpression or knockout of one *AtLACS2* ortholog in *B. napus* impacted seed oil content (Ding et al., 2020). These results demonstrate that LACSs display conserved roles in TAG synthesis in the identified plant species, but we cannot ignore that TAG FA compositions vary among species. For example, oleic acids are the predominant FA constituent of TAG in rapeseed oil, whereas linoleic acids dominate *Arabidopsis* seed oil (Guo et al., 2019; Ding et al., 2020). As such, to precisely interpret the function of each LACS in TAG biosynthesis, multiple factors need to be considered, including plant species, substrate preference, tissue expression pattern, subcellular localization, and protein-level interactions with other enzymes involved in TAG synthesis.

## Long-Chain Acs Mutations Cause Pleotropic Phenotypes During Plant Development

Owing to the functional redundancy of LACS isoforms in *Arabidopsis*, most single *atlacs* mutants display no visible phenotypes, but *atlacs2* is one exception. Given LACS2’s primary function in cutin synthesis and ubiquitous pattern of expression, its deficiency leads to multiple defects in plant growth including reduced leaf size, overall plant size and seed sets, and lower rates of germination and seedling establishment (Schnurr et al., 2004). To further comprehend the roles of *Arabidopsis LACS* genes in plant growth and development, several research groups have generated higher-order mutants defective in the expression of multiple *AtLACS* genes. These mutants display much more severe phenotypes than their corresponding parental lines (Fulda et al., 2004; Weng et al., 2010; Zhao et al., 2019). For example, *atlacs4*, *atlacs8*, and *atlacs9* single mutants do not exhibit visible phenotypes; however, *atlacs4 atlacs8* and *atlacs4 atlacs9* double mutants display phenotypes reminiscent of the *atlacs2* single mutant (Jessen et al., 2015; Zhao et al., 2019). Furthermore, the *atlacs4 atlacs8 atlacs9* triple mutant exhibits lethality (Zhao et al., 2019), suggesting that LACS activity is vital for plant growth. Additionally, we noticed that *atlacs1atlacs2* double mutant displays organ fusion (Weng et al., 2010), which resembles the phenotype of several cuticle-deficient mutants such as *lacerata*, *bodyguard*, and *atp-binding cassette proteins g11* and *g13* (Ingram and Nawrath, 2017). These studies indicate that *LACS* genes participate in the formation of organ boundaries.

Besides the aforementioned phenotypes, male fertility is also closely related with LACS activity. AtLACS1 and AtLACS4 are reported to be associated with the synthesis of both wax and pollen lipids (tryphine). Tryphine is a mixture of very-long-chain lipids including alkanes, alkenes, primary alcohols, secondary alcohols, aldehydes, and free FAs. Simultaneous suppression of AtLACS1 and AtLACS4 activity led to significant reduction in wax as well as tryphine lipids resulting in conditional male sterility, albeit could be rescued by high humidity (Jessen et al., 2011). However, it seems that AtLACS1 and AtLACS4 have opposing roles in the tryphine synthesis given that tryphine levels are apparently decreased in the *atlacs1* mutant but are dramatically increased in the *atlacs4* mutant (Jessen et al., 2011). It is unclear why high levels of tryphine accumulate in the *atlacs4* mutant. The authors proposed that the accumulation of tryphine in *atlacs4* mutant might be due to the defective lipid transport from tapetal cells to developing pollen grains (Jessen et al., 2011). The roles of LACS in male fertility seem to be conserved, since GhACS1 in cotton, an ortholog of AtLACS4 and AtLACS5 is preferentially expressed in developing anther, and cotton plants with reduced GhACS1 activity produce defective microspores (Wang and Li, 2009).

Different from the roles of those AtLACS isoforms involved in lipid synthesis, AtLACS6 and AtLACS7 are peroxisome-located and involved in lipid degradation, providing energy for seed germination and other energy-requiring physiological processes (Shockey et al., 2002). Thus, simultaneous deficiency of both genes delays post-germination seedling growth due to the lack of sufficient energy (Fulda et al., 2004).

## Long-Chain Acss Are Associated With Stress Resistances Including Drought, Hypoxia, and Biotic Stresses

Cuticle forms surface barriers against biotic and abiotic stresses. As described above, several LACSs have been identified to be involved in the synthesis cuticular lipids and thus are vital for plant stress resistance. The main function of cuticle is to prevent non-stomatal water loss. To date, several *Arabidopsis LACS* single or double mutants including *atlacs2*, *atlacs1 atlacs2*, *atlacs4 atlacs8*, and *atlacs4 atlacs9* are reported to show increased cuticle permeability, increased water loss, and the hypersensitivity to drought (Bessire et al., 2007; Lü et al., 2009; Weng et al., 2010; Zhao et al., 2019). Moreover, owing to functional redundancy, higher-order *atlacs* mutants often show increased sensitivity to drought. The *atlacs1 atlacs2 atlacs4* triple mutant is a prime example. The rosette water loss rate of the *atlacs1 atlacs2 atlacs4* triple mutant exceeds that of single or double mutants (Jessen et al., 2015; Zhao et al., 2019). Furthermore, the role of LACSs in drought tolerance has been described in species other than *Arabidopsis*. Zhang et al. (2020a, b) found that ectopic expression of two *LACSs* from apple (*MdLACS2* and *MdLACS4*) in *Arabidopsis* decreased epidermal permeability, reduced water loss, and enhanced the resistance of transgenic plants to drought and salt stress. In another study, Zhang et al. (2018) demonstrated that callus transformed with *MdLACS1* showed enhanced tolerance to PEG, NaCl, and ABA treatments. These results indicate that the roles of LACS activity in cuticle synthesis are indispensable for plant resistance to drought.

Long-chain ACS activity is also closely related with hypoxia response. RAP2.12, a member of subgroup VII ETHYLENE-RESPONSE FACTOR (ERFVII) transcription factors, is a key regulator of hypoxic gene expression (Licausi et al., 2011; Schmidt et al., 2018; Schmidt and van Dongen, 2019; Xie et al., 2020). Under normoxic conditions, this protein is trapped in the PM through direct binding with its interacting partners, ACYL-CoA BINDING PROTEINs (ACBPs). Under hypoxic conditions, RAP2.12 is released from the PM and translocated to the nucleus to induce the expression of hypoxia responsive genes (Licausi et al., 2011; Schmidt et al., 2018; Schmidt and van Dongen, 2019; Xie et al., 2020). Acyl-CoAs are reported to act as signals triggering the dissociation of RAP2.12 from ACBPs upon hypoxia stimulus (Schmidt et al., 2018; Xie et al., 2020). Acyl-CoA synthesis requires the participation of LACSs enzymes. Several AtLACSs have been identified to be related with the translocation of RAP2.12 (Schmidt et al., 2018; Xie et al., 2020). Simultaneous mutation in *AtLACS4* and *AtLACS9* resulted in the accumulation of high levels of oleoyl-CoA, which triggers the accumulation of RAP2.12 in nucleus, thus inducing high expression of hypoxia response genes (Schmidt et al., 2018). Mutations in the *AtLACS2* gene are also reported to induce hypoxia responses via increased levels of polyunsaturated linolenoyl-CoA, which promotes the translocation of RAP2.12 to the nucleus (Xie et al., 2020). Not surprisingly, *atlacs* mutants exhibit increased sensitivity to hypoxia (Schmidt et al., 2018; Xie et al., 2020), whereas *AtLACS2* overexpression enhanced tolerance to hypoxia (Xie et al., 2020). Taken together, LACS activity is required for regulating ERF-VII-mediated hypoxia signaling.

Long-chain ACS activity is not only required for plant resistance to abiotic stresses but also important for plant responses to biotic stress. For example, *atlacs2* mutants display strong resistance to infection by a necrotrophic fungus, *Botrytis cinerea* (Bessire et al., 2007). The author proposed that loss of *AtLACS2* function increased cuticle permeability and thus might facilitate the diffusion of signals and effector molecules across the cuticle, which triggers a protection mechanism against the attack of *B. cinerea* (Bessire et al., 2007). But different from the response to *B. cinerea*, *AtLACS2* loss of function increases susceptibility to avirulent *Pseudomonas syringae* (Tang et al., 2007). Possibly, the *atlacs2* mutant has reduced stomatal ledges like other cuticle mutants, allowing more *Pseudomonas* swim through the stomata and enter the leaf (Lü et al., 2009). Taken together, the alteration of LACS activity changes the cuticle structure that ultimately affects plant physiological responses.

## Regulation of Long-Chain Acs Activity

Long-chain ACSs provide various acyl-CoA pools for formation of the cuticle layer, the synthesis of membrane lipids and storage TAG, and β-oxidation. *LACSs* are known to be regulated at multiple levels. Until recently, more focus has been given to the transcriptional regulation of *LACS* genes. During cuticle biosynthesis, *AtLACSs* together with other cuticle-related genes are regulated by two types of transcription factors that have antagonistic functions. Transcription factors such as WAX INDUCER1/SHINE (WIN1), MYB16, MYB30, and MYB106 are identified to act as positive regulators of *AtLACS2* (Kannangara et al., 2007; Raffaele et al., 2008; Oshima et al., 2013). *AtLACS2* expression is also negatively regulated by DECREASE WAX BIOSYNTHESIS1 (DEWAX1) and DEWAX2, two members of AP2/ethylene response element-binding factor (ERF)-type transcription factor family, which directly bind to the promoter region of *AtLACS2* (Go et al., 2014; Kim et al., 2018). Just like DEWAXs, AtMYB41, one member of R2R3-MYB transcription factor, is also identified to be a negative regulator of *AtLACS2*, though it is unknown if their interaction is direct (Cominelli et al., 2008). Besides these transcription factors, two E3 ligases, HISTONE MONOUBIQUITINATION 1 and 2 (HUB1 and HUB2), are also reported to transcriptionally activate the expression of several cutin-related genes including *AtLACS2* through remodeling chromatin structure (Menard et al., 2014). Taken together, the *LACS* genes are transcriptionally coregulated by multiple factors. These mechanisms of regulation appear to be conserved in other plant species. For example, PeSHN1, one poplar homolog of SHN1, is also identified to positively regulate the expression of *LACS2* in poplar (Meng et al., 2019), and MdMYB30 is found to activate the expression of *MdLACS2* in apple (Zhang et al., 2019).

Long-chain ACS activity can also be post-transcriptionally regulated. With the development of deep-sequencing technology, a large number of microRNAs have been identified in diverse plant species. Many of these microRNAs play important regulatory roles during plant development. Genes related with FAs synthesis were found to be regulated by microRNA in oil palm, including orthologs of *AtLACS4* and *AtLACS9*, which were identified to be targeted by eg-miR444b and eg-miR397, respectively (Zheng et al., 2019). The regulation of *LACS* genes by microRNAs may also be a mechanism utilized by other plant species. The plethora of whole-genome sequences available from diverse plant species will facilitate studies of this nature.

Long-chain ACS activity is also regulated at the level of translation. Xu et al. (2019) reported that D238 in AtLACS9 is predicted to have been positively selected during evolution, which is naturally replaced by glutamic acid in other plant species. This substitution is identified to enhance enzyme activity possibly by creating a more favorable enzyme conformation, suggesting that some key amino acids are vital for enzyme activity. Site-directed mutagenesis or domain swapping or deletion will help to advance our understanding of LACS catalytic mechanism. In our work, we found that one E3 ligase (known as CER9) genetically interacts with AtLACSs since its deficiency shows additive effects with *AtLACS1* or *AtLACS2* on wax or cutin synthesis (Lü et al., 2012). We hypothesize that CER9 physically interacts with AtLACSs proteins, trapping them in the ER to maintain their normal activity or that CER9 targets a negative regulator of AtLACS for degradation. However, we cannot also eliminate the possibility that CER9 might target a thioesterase that would hydrolyze VLCFA-CoAs to VLCFAs. Clearly, experimental validation of these hypotheses is required. In addition to these intrinsic regulators, cellular energy status is also closely related with LACS activity. As such, energy-limited conditions will inevitably affect LACS activity. For example, LACS activity is apparently affected by hypoxia, a condition that limits energy that would be produced by aerobic respiration (Schmidt et al., 2018).

## Conclusion and Perspectives

Long-chain ACSs target LCFAs or VLCFAs to providing acyl-CoA substrates for lipid synthesis and degradation processes vital for plant normal growth. Herein, we summarize their tissue-specific expression patterns and subcellular localizations and provide a summary of their specific roles in different metabolic pathways. Lastly, we describe mechanisms by which LACS is regulated. Though great progress has been made on comprehending LACS function, a number of questions remain to be answered.

As a case in point, a function for LACS in regulating suberin biosynthesis remains unproven. Suberin comprises hydroxylated FAs, dioic acids, fatty alcohols, hydroxycinnamic acids, and glycerol (Philippe et al., 2020), with chemical analogy to cutin monomers, suggesting that some enzymes may be used for both cutin and suberin biosynthesis. Moreover, it is reported that the overexpression or knockdown of cutin-related genes often changes suberin profiles (Philippe et al., 2020). To date, the role of AtLACSs in cutin synthesis is well characterized, but their role in suberin synthesis is less known. Based on *in vitro* enzyme assays, AtLACS1, AtLACS2, and AtLACS4 are likely to participate in suberin biosynthesis (Philippe et al., 2020), but no direct evidence of this function has been obtained. Additionally, Genevestigator data show that *AtLACS2*, *AtLACS3*, and *AtLACS9* are specifically expressed in the root endodermis where suberin distributes, implying a function in suberin synthesis. To verify these speculations, it will be necessary to determine the suberin composition and content of these *LACS* mutants by biochemical methods. This will help to provide a more complete understanding of the roles of LACS members in lipid metabolism.

Long-chain ACS proteins are known to have dual functions, acting as enzymes that activate LCFAs and VLCFAs, but also as lipid transporters. Most studies have focused on the role of LACS in activating Fas; however, a function for LACS in lipid transport *in planta* is less understood. Pulsifer et al. (2012) reported that the ectopic expression of *AtLACS1*, *AtLACS2*, and *AtLACS3* in yeast complements the phenotype of a yeast *fat1D* mutant deficient in both very-long-chain ACS activity and exogenous FA uptake. It has also been proposed that AtLACS4 and AtLACS9 mediate the import of FAs generated in the ER into plastids according to the analysis of metabolic products in *lacs* mutants (Jessen et al., 2015). These studies cannot fully elaborate the role of LACS in lipid transport. Thus, comprehending LACS function in lipid transport *in planta* may better define subcellular lipid channeling routes and comprehensively elaborate the roles of LACS in plant lipid metabolism.

Though the function of LACS proteins is well identified in *Arabidopsis*, more studies are needed to understand the functional divergence of LACS during evolution and breeding. As shown in Table 1, LACSs usually display variable substrate preferences since FA constituents often vary among different species. For example, TpLACSA in *T. pseudonana*, which shares high sequence identity with AtLACS7 and AtLACS6, preferentially catalyzes the formation of acyl-CoAs from polyunsaturated FAs (PUFAs) including arachidonic acid (20:4), eicosapentaenoic acid (20:5), and docosahexaenoic acid (22:6) (Table 1). However, these PUFAs are not present in *Arabidopsis* and thus are not potential preferred substrates of AtLACS6 and AtLACS7. Additionally, LACS copy number varies greatly among the genomes of different species (Shockey et al., 2002; Zhang et al., 2018; Ding et al., 2020). Thirty-four LACS genes are found in *B. napus*, 11 LACS orthologs are present in *M. domestica*, and only nine LACS genes are found in *Arabidopsis*. These LACSs display different patterns of expression indicative of functional divergence during evolution. Thus, extensive research in species with divergent lipid compositions will expand our knowledge of LACS function in plants and provide a more comprehensive understanding of lipid metabolism across the plant kingdom.

## Author Contributions

HZ wrote the manuscript. DK and SL made critical revision. All authors contributed to the article and approved the submitted version.

## Conflict of Interest

The authors declare that the research was conducted in the absence of any commercial or financial relationships that could be construed as a potential conflict of interest.

## Footnotes

1 http://efp.ucr.edu/cgi-bin/relative.cgi

## References

[B1] Aznar-Moreno J. A. Venegas Caleron M. Martinez-Force E. Garces R. Mullen R. Gidda S. K. (2014). Sunflower (*Helianthus annuus*) long-chain acyl-coenzyme A synthetases expressed at high levels in developing seeds. *Physiol. Plant.* 150 363–373. 10.1111/ppl.12107 24102504

[B2] Bessire M. Chassot C. Jacquat A. C. Humphry M. Borel S. Petetot J. M. (2007). A permeable cuticle in Arabidopsis leads to a strong resistance to *Botrytis cinerea*. *EMBO J.* 26 2158–2168. 10.1038/sj.emboj.7601658 17396154 PMC1852784

[B3] Cominelli E. Sala T. Calvi D. Gusmaroli G. Tonelli C. (2008). Over-expression of the Arabidopsis AtMYB41 gene alters cell expansion and leaf surface permeability. *Plant J.* 53 53–64. 10.1111/j.1365-313X.2007.03310.x 17971045

[B4] Ding L. N. Gu S. L. Zhu F. G. Ma Z. Y. Li J. Li M. (2020). Long-chain acyl-CoA synthetase 2 is involved in seed oil production in *Brassica napus*. *BMC Plant Biol.* 20:21. 10.1186/s12870-020-2240-x 31931712 PMC6958636

[B5] Fich E. A. Segerson N. A. Rose J. K. C. (2016). The plant polyester Cutin: biosynthesis, structure, and biological roles. *Annu. Rev. Plant Biol.* 67 207–233. 10.1146/annurev-arplant-043015-111929 26865339

[B6] Fulda M. Schnurr J. Abbadi A. Heinz E. Browse J. (2004). Peroxisomal Acyl-CoA synthetase activity is essential for seedling development in *Arabidopsis thaliana*. *Plant Cell* 16 394–405. 10.1105/tpc.019646 14742880 PMC341912

[B7] Fulda M. Shockey J. Werber M. Wolter F. P. Heinz E. (2002). Two long-chain acyl-CoA synthetases from *Arabidopsis thaliana* involved in peroxisomal fatty acid beta-oxidation. *Plant J.* 32 93–103. 10.1046/j.1365-313X.2002.01405.x 12366803

[B8] Go Y. S. Kim H. Kim H. J. Suh M. C. (2014). Arabidopsis Cuticular Wax biosynthesis is negatively regulated by the DEWAX gene encoding an AP2/ERF-type transcription factor. *Plant Cell* 26 1666–1680. 10.1105/tpc.114.123307 24692420 PMC4036578

[B9] Grevengoed T. J. Klett E. L. Coleman R. A. (2014). Acyl-CoA metabolism and partitioning. *Annu. Rev. Nutr.* 34 1–30. 10.1146/annurev-nutr-071813-105541 24819326 PMC5881898

[B10] Guo Z. H. Ye Z. W. Haslam R. P. Michaelson L. V. Napier J. A. Chye M. L. (2019). Arabidopsis cytosolic acyl-CoA-binding proteins function in determining seed oil composition. *Plant Direct* 3:e00182. 10.1002/pld3.182 31844833 PMC6892995

[B11] He X. Chen G. Q. Kang S. T. Mckeon T. A. (2007). *Ricinus communis* contains an acyl-CoA synthetase that preferentially activates ricinoleate to its CoA thioester. *Lipids* 42 931–938. 10.1007/s11745-007-3090-0 17680295

[B12] Ingram G. Nawrath C. (2017). The roles of the cuticle in plant development: organ adhesions and beyond. *J. Exp. Bot.* 68 5307–5321. 10.1093/jxb/erx313 28992283

[B13] Jessen D. Olbrich A. Knufer J. Kruger A. Hoppert M. Polle A. (2011). Combined activity of *LACS1* and *LACS4* is required for proper pollen coat formation in Arabidopsis. *Plant J.* 68 715–726. 10.1111/j.1365-313X.2011.04722.x 21790813

[B14] Jessen D. Roth C. Wiermer M. Fulda M. (2015). Two activities of long-chain acyl-coenzyme A synthetase are involved in lipid trafficking between the endoplasmic reticulum and the plastid in Arabidopsis. *Plant Physiol.* 167 351–366. 10.1104/pp.114.250365 25540329 PMC4326746

[B15] Kannangara R. Branigan C. Liu Y. Penfield T. Rao V. Mouille G. (2007). The transcription factor WIN1/SHN1 regulates Cutin biosynthesis in *Arabidopsis thaliana*. *Plant Cell* 19 1278–1294. 10.1105/tpc.106.047076 17449808 PMC1913754

[B16] Kim H. Go Y. S. Suh M. C. (2018). DEWAX2 transcription factor negatively regulates cuticular wax biosynthesis in Arabidopsis leaves. *Plant Cell Physiol.* 59 966–977. 10.1093/pcp/pcy033 29425344

[B17] Kitajima-Koga A. Baslam M. Hamada Y. Ito N. Taniuchi T. Takamatsu T. Oikawa K. Kaneko K. Mitsui T. (2020). Functional Analysis of Rice Long-Chain Acyl-CoA Synthetase 9 (*OsLACS9*) in the Chloroplast Envelope Membrane. *Int. J. Mol. Sci.* 21:2223. 10.3390/ijms21062223 PMC7139535 32210132

[B18] Lee S. B. Suh M. C. (2013). Recent advances in cuticular wax biosynthesis and its regulation in *Arabidopsis*. *Mol. Plant* 6 246–249. 10.1093/mp/sss159 23253604

[B19] Li-Beisson Y. Shorrosh B. Beisson F. Andersson M. X. Arondel V. Bates P. D. (2013). Acyl-lipid metabolism. *Arabidopsis Book* 11:e0161. 10.1199/tab.0161 23505340 PMC3563272

[B20] Licausi F. Kosmacz M. Weits D. A. Giuntoli B. Giorgi F. M. Voesenek L. A. (2011). Oxygen sensing in plants is mediated by an N-end rule pathway for protein destabilization. *Nature* 479 419–422. 10.1038/nature10536 22020282

[B21] Lü S. Song T. Kosma D. K. Parsons E. P. Rowland O. Jenks M. A. (2009). Arabidopsis CER8 encodes LONG-CHAIN ACYL-COA SYNTHETASE 1 (LACS1) that has overlapping functions with LACS2 in plant wax and cutin synthesis. *Plant J.* 59 553–564. 10.1111/j.1365-313X.2009.03892.x 19392700

[B22] Lü S. Zhao H. Des Marais D. L. Parsons E. P. Wen X. Xu X. (2012). Arabidopsis ECERIFERUM9 involvement in cuticle formation and maintenance of plant water status. *Plant Physiol.* 159 930–944. 10.1104/pp.112.198697 22635115 PMC3387718

[B23] Menard R. Verdier G. Ors M. Erhardt M. Beisson F. Shen W. H. (2014). Histone H2B monoubiquitination is involved in the regulation of cutin and wax composition in *Arabidopsis thaliana*. *Plant Cell Physiol.* 55 455–466. 10.1093/pcp/pct182 24319075

[B24] Meng S. Cao Y. Li H. Bian Z. Wang D. Lian C. Yin W. Xia X. (2019). PeSHN1 regulates water-use efficiency and drought tolerance by modulating wax biosynthesis in poplar. *Tree Physiol.* 39 1371–1386. 10.1093/treephys/tpz033 30938421

[B25] Oshima Y. Shikata M. Koyama T. Ohtsubo N. Mitsuda N. Ohme-Takagi M. (2013). MIXTA-like transcription factors and WAX INDUCER1/SHINE1 coordinately regulate cuticle development in Arabidopsis and Torenia fournieri. *Plant Cell.* 25 1609–1624. 10.1105/tpc.113.110783 23709630 PMC3694695

[B26] Philippe G. Sorensen I. Jiao C. Sun X. Fei Z. Domozych D. S. (2020). Cutin and suberin: assembly and origins of specialized lipidic cell wall scaffolds. *Curr. Opin. Plant Biol.* 55 11–20. 10.1016/j.pbi.2020.01.008 32203682

[B27] Pulsifer I. P. Kluge S. Rowland O. (2012). Arabidopsis long-chain acyl-CoA synthetase 1 (LACS1), LACS2, and LACS3 facilitate fatty acid uptake in yeast. *Plant Physiol. Biochem.* 51 31–39. 10.1016/j.plaphy.2011.10.003 22153237

[B28] Raffaele S. Vailleau F. Leger A. Joubes J. Miersch O. Huard C. (2008). A MYB transcription factor regulates very-long-chain fatty acid biosynthesis for activation of the hypersensitive cell death response in *Arabidopsis*. *Plant Cell* 20 752–767. 10.1105/tpc.107.054858 18326828 PMC2329921

[B29] Schmidt R. R. Fulda M. Paul M. V. Anders M. Plum F. Weits D. A. (2018). Low-oxygen response is triggered by an ATP-dependent shift in oleoyl-CoA in Arabidopsis. *Proc. Natl. Acad. Sci. U.S.A.* 115 E12101–E12110. 10.1073/pnas.1809429115 30509981 PMC6304976

[B30] Schmidt R. R. van Dongen J. T. (2019). The ACBP1-RAP2.12 signalling hub: a new perspective on integrative signalling during hypoxia in plants. *Plant Signal. Behav.* 14 e1651184. 10.1080/15592324.2019.1651184 31397636 PMC6768276

[B31] Schnurr J. Shockey J. Browse J. (2004). The acyl-CoA synthetase encoded by LACS2 is essential for normal cuticle development in Arabidopsis. *Plant Cell* 16 629–642. 10.1105/tpc.017608 14973169 PMC385277

[B32] Schnurr J. A. Shockey J. M. De Boer G. J. Browse J. A. (2002). Fatty acid export from the chloroplast. Molecular characterization of a major plastidial acyl-coenzyme A synthetase from Arabidopsis. *Plant Physiol* 129 1700–1709. 10.1104/pp.003251 12177483 PMC166758

[B33] Shockey J. Browse J. (2011). Genome-level and biochemical diversity of the acyl-activating enzyme superfamily in plants. *Plant J*. 66 143–160. 10.1111/j.1365-313X.2011.04512.x 21443629

[B34] Shockey J. M. Fulda M. S. Browse J. A. (2002). Arabidopsis contains nine long-chain acyl-coenzyme a synthetase genes that participate in fatty acid and glycerolipid metabolism. *Plant Physiol.* 129 1710–1722. 10.1104/pp.003269 12177484 PMC166759

[B35] Soupene E. Kuypers F. A. (2008). Mammalian long-chain acyl-CoA synthetases. *Exp. Biol. Med.* 233 507–521. 10.3181/0710-MR-287 18375835 PMC3377585

[B36] Suh M. C. Samuels A. L. Jetter R. Kunst L. Pollard M. Ohlrogge J. (2005). Cuticular lipid composition, surface structure, and gene expression in Arabidopsis stem epidermis. *Plant Physiol.* 139 1649–1665. 10.1104/pp.105.070805 16299169 PMC1310549

[B37] Tang D. Simonich M. T. Innes R. W. (2007). Mutations in LACS2, a long-chain acyl-coenzyme A synthetase, enhance susceptibility to avirulent *Pseudomonas syringae* but confer resistance to *Botrytis cinerea* in Arabidopsis. *Plant Physiol.* 144 1093–1103. 10.1104/pp.106.094318 17434992 PMC1914183

[B38] Tonon T. Qing R. Harvey D. Li Y. Larson T. R. Graham I. A. (2005). Identification of a long-chain polyunsaturated fatty acid acyl-coenzyme A synthetase from the diatom *Thalassiosira pseudonana*. *Plant Physiol.* 138 402–408. 10.1104/pp.104.054528 15821149 PMC1104193

[B39] Wang X. L. Li X. B. (2009). The GhACS1 gene encodes an acyl-CoA synthetase which is essential for normal microsporogenesis in early anther development of cotton. *Plant J.* 57 473–486. 10.1111/j.1365-313X.2008.03700.x 18826432

[B40] Weng H. Molina I. Shockey J. Browse J. (2010). Organ fusion and defective cuticle function in a *lacs1 lacs2* double mutant of *Arabidopsis*. *Planta* 231 1089–1100. 10.1007/s00425-010-1110-4 20237894

[B41] Xiao Z. Li N. Wang S. Sun J. Zhang L. Zhang C. (2019). Genome-wide identification and comparative expression profile analysis of the long-chain Acyl-CoA synthetase (LACS) gene family in two different oil content cultivars of *Brassica napus*. *Biochem. Genet.* 57 781–800. 10.1007/s10528-019-09921-5 31011871

[B42] Xie L. J. Tan W. J. Yang Y. C. Tan Y. F. Zhou Y. Zhou D. M. (2020). Long-Chain acyl-CoA synthetase LACS2 contributes to submergence tolerance by modulating cuticle permeability in Arabidopsis. *Plants* 9:262. 10.3390/plants9020262 32085442 PMC7076686

[B43] Xu Y. Caldo K. M. P. Holic R. Mietkiewska E. Ozga J. Rizvi S. M. (2019). Engineering Arabidopsis long-chain acyl-CoA synthetase 9 variants with enhanced enzyme activity. *Biochem. J.* 476 151–164. 10.1042/BCJ20180787 30559328

[B44] Xu Y. Holic R. Li D. Pan X. Mietkiewska E. Chen G. (2018). Substrate preferences of long-chain acyl-CoA synthetase and diacylglycerol acyltransferase contribute to enrichment of flax seed oil with alpha-linolenic acid. *Biochem. J.* 475 1473–1489. 10.1042/BCJ20170910 29523747

[B45] Yang X. Zhao H. Kosma D. K. Tomasi P. Dyer J. M. Li R. (2017). The acyl desaturase CER17 is involved in producing wax unsaturated primary alcohols and cutin monomers. *Plant Physiol.* 173 1109–1124. 10.1104/pp.16.01956 28069670 PMC5291053

[B46] Yu L. Tan X. Jiang B. Sun X. Gu S. Han T. (2014). A peroxisomal long-chain acyl-CoA synthetase from Glycine max involved in lipid degradation. *PLoS One* 9:e100144. 10.1371/journal.pone.0100144 24992019 PMC4081121

[B47] Zhang C. L. Hu X. Zhang Y. L. Liu Y. Wang G. L. You C. X. (2020a). An apple long-chain acyl-CoA synthetase 2 gene enhances plant resistance to abiotic stress by regulating the accumulation of cuticular wax. *Tree Physiol.* 40 1450–1465. 10.1093/treephys/tpaa079 32578855

[B48] Zhang C. L. Mao K. Zhou L. J. Wang G. L. Zhang Y. L. Li Y. Y. (2018). Genome-wide identification and characterization of apple long-chain Acyl-CoA synthetases and expression analysis under different stresses. *Plant Physiol. Biochem.* 132 320–332. 10.1016/j.plaphy.2018.09.004 30248518

[B49] Zhang C. L. Zhang Y. L. Hu X. Xiao X. Wang G. L. You C. X. (2020b). An apple long-chain acyl-CoA synthetase, MdLACS4, induces early flowering and enhances abiotic stress resistance in Arabidopsis. *Plant Sci.* 297:110529. 10.1016/j.plantsci.2020.110529 32563467

[B50] Zhang Y. L. Zhang C. L. Wang G. L. Wang Y. X. Qi C. H. Zhao Q. (2019). The R2R3 MYB transcription factor MdMYB30 modulates plant resistance against pathogens by regulating cuticular wax biosynthesis. *BMC Plant Biol.* 19:362. 10.1186/s12870-019-1918-4 31426743 PMC6700842

[B51] Zhao L. Haslam T. M. Sonntag A. Molina I. Kunst L. (2019). Functional overlap of long-chain Acyl-CoA synthetases in Arabidopsis. *Plant Cell Physiol.* 60 1041–1054. 10.1093/pcp/pcz019 30715495

[B52] Zhao L. Katavic V. Li F. Haughn G. W. Kunst L. (2010). Insertional mutant analysis reveals that long-chain acyl-CoA synthetase 1 (LACS1), but not LACS8, functionally overlaps with LACS9 in Arabidopsis seed oil biosynthesis. *Plant J.* 64 1048–1058. 10.1111/j.1365-313X.2010.04396.x 21143684

[B53] Zheng Y. Chen C. Liang Y. Sun R. Gao L. Liu T. (2019). Genome-wide association analysis of the lipid and fatty acid metabolism regulatory network in the mesocarp of oil palm (*Elaeis guineensis* Jacq.) based on small noncoding RNA sequencing. *Tree Physiol.* 39 356–371. 10.1093/treephys/tpy091 30137626

